# Metabolomic analysis of the food-borne pathogen *Campylobacter jejuni*: application of direct injection mass spectrometry for mutant characterisation

**DOI:** 10.1007/s11306-014-0644-z

**Published:** 2014-03-06

**Authors:** Robert M. Howlett, Matthew P. Davey, W. Paul Quick, David J. Kelly

**Affiliations:** 1 Department of Molecular Biology and Biotechnology, The University of Sheffield, Firth Court, Western Bank, Sheffield, S10 2TN UK; 2 Department of Plant Sciences, University of Cambridge, Downing Street, Cambridge, CB2 3EA UK; 3 Department of Animal and Plant Sciences, The University of Sheffield, Western Bank, Sheffield, S10 2TN UK; 4 Present Address: Department of Biology, University of York, York, North Yorkshire YO10 5DD UK

**Keywords:** *Campylobacter jejuni*, DIMS, Metabolism, *aspA*, *aspB*, *sdaA*

## Abstract

*Campylobacter jejuni* is the most frequent cause of human food-borne bacterial gastroenteritis but its physiology and biochemistry are poorly understood. Only a few amino-acids can be catabolised and these are known to be important for host colonization. Here we have established methods for rapid high throughput analyses of global metabolism in *C. jejuni* using direct injection mass spectrometry (DIMS) to compare metabolite fingerprints of wild-type and mutant strains. Principal component analyses show that the metabolic fingerprint of mutants that have a genomic deletion in genes for key amino-acid catabolic enzymes (either *sdaA,* serine dehydratase; *aspA,* aspartase or *aspB,* aspartate:glutamate transaminase) can easily be distinguished from the isogenic parental strain. Assignment of putative metabolites showed predictable changes directly associated with the particular metabolic lesion in these mutants as well as more extensive changes in the *aspA* mutant compared to the *sdaA* or *aspB* strains. Further analyses of a *cj0150c* mutant strain, which has no obvious phenotype, suggested a role for Cj0150 in the conversion of cystathionine to homocysteine. Our results show that DIMS is a useful technique for probing the metabolism of this important pathogen and may help in assigning function to genes encoding novel enzymes with currently unknown metabolic roles.

**Electronic supplementary material:**

The online version of this article (doi:10.1007/s11306-014-0644-z) contains supplementary material, which is available to authorized users.

## Introduction

*Campylobacter jejuni* is the leading cause of human bacterial gastroenteritis in the western world (Scallan et al. 2011) with over 400 million reported cases each year world wide (Ruiz-Palacios 2007), resulting in a large economic burden (Batz et al. 2012). The bacterium is commensal in the intestines of chickens and most infections arise from consumption of contaminated poultry meat. The physiology and metabolism of *C. jejuni* is less well understood compared to other food-borne pathogens. A limited ability to metabolise sugars along with the absence of phosphofructokinase (Parkhill et al. 2000) results in a major requirement for non-carbohydrate derived carbon sources in *C. jejuni* (Velayudhan and Kelly 2002). Previous studies have shown that only a limited range of amino acids and certain TCA cycle intermediates, are important carbon sources for *C. jejuni* (Velayudhan et al. 2004; Guccione et al. 2008; Hofreuter et al. 2008). The ability to use aspartate, glutamate (Leon-Kempis et al. 2006; Guccione et al. 2008), serine (Velayudhan et al. 2004; Hofreuter et al. 2012) and proline (Guccione et al. 2008; Hofreuter et al. 2008, 2012) is characteristic of most strains, with mutations in *aspA* (aspartase) *aspB* (aspartate:glutamate aminotransferase) and *sdaA* (serine dehydratase) genes found to result in an inability to catabolise these amino acids and defects in the colonisation of chicken and mouse animal models (Velayudhan et al. 2004; Guccione et al. 2008; Hofreuter et al. 2008, 2012).

Global metabolomic approaches have not previously been used to study the central carbon metabolism of *C. jejuni*. Although nuclear magnetic resonance methods have been used for this type of application (e.g. Raamsdonk et al. 2001), sensitivity of metabolite detection remains an issue. A potentially useful technique that has previously been used for high throughput fingerprinting is automated electrospray ionisation time of flight mass spectrometry (ESI-TOF MS) (Dunn et al. 2005). ESI-TOF MS is a method that enables rapid sample runtime and relatively simple mass spectra for complex samples, albeit at the expense of accurate mass identification and quantification (Dunn et al. 2005). Although originally utilised for high-throughput clinical screening there have also been several microbial applications of this technique, often in bacterial strain discrimination (Goodacre et al. 1998; Vaidyanathan et al. 2001), including MALDI-TOF mass spectrometry based identification of *Campylobacter* species (Bessède et al. 2011). The possibility of utilising Direct Injection ESI-TOF MS (DIMS) as a means of rapidly analysing gene knockout mutant libraries has also been suggested, with the major advantage of minimal sample preparation and processing. Work by Kaderbhai et al. (2003) found DIMS to be capable of discriminating between tryptophan metabolism mutants in *Escherichia coli* and ESI-TOF MS has been suggested as an advantageous methodology for assigning unknown gene function (Castrillo et al. 2003).

In this study, previously described *C. jejuni* null mutants in *aspA, aspB* (Guccione et al. 2008) and *sdaA* (Velayudhan et al. 2004) have been analysed by DIMS in order to assess whether the metabolic lesions in these strains can be confirmed by their metabolic fingerprints and whether other changes in the metabolome can be inferred from putative metabolite identifications. The relative abundances of key metabolites have been found to show the changes expected in these mutants and the techniques have also been applied to a mutant in *cj0150c* (Guccione et al. 2008), a gene of unknown function, to generate a hypothesis for the role of the encoded aminotransferase enzyme, Cj0150. Our results suggest that non-targeted, high sensitivity, low mass accuracy direct injection techniques can be useful for probing the metabolism of poorly understood pathogens like *C. jejuni* and for generating hypotheses for the function of genes with currently unknown roles.

## Materials and methods

### Bacterial strains, media and growth conditions

The strains used in this study were *C. jejuni* NCTC 11168 wild-type and a set of isogenic mutants carrying null mutations in the *sdaA, aspA, aspB* and *cj0150c* genes. These mutants have been fully described in previous publications (Velayudhan et al. 2004; Guccione et al. 2008) and were constructed by the insertion of antibiotic resistance cassettes into the appropriate genes. The mutants were obtained from the Kelly lab culture collection and verified by PCR analysis with gene specific primers. All bacterial strains were routinely grown on Columbia agar containing 5 % (v/v) lysed horse blood and 10 μg mL^−1^ amphotericin B and vancomycin at 37 °C under microaerobic conditions [10 % (v/v) O_2_, 5 % (v/v) CO_2_ and 85 % (v/v) N_2_] in a MACS growth cabinet (Don Whitley Scientific, Shipley, UK). For mutant strain analysis five biological replicate cultures were grown alongside five biological replicates of the isogenic parent strain in 25 mL volumes of brain heart infusion (BHI) (Oxoid) containing 5 % (v/v) foetal calf serum (FCS; GIBCO) under microaerobic conditions with continuous shaking at 180 rpm.

### Extraction of *C. jejuni* metabolites

Cells taken for metabolite extraction were harvested by centrifugation (15,871 *g*, 1 min, 20 °C), the supernatants removed and the cell pellets flash frozen in liquid nitrogen. This decreased the possibility of cell lysis resulting in metabolites entering the supernatant and hence increased the number of measureable metabolites within the cells. Cell density was normalised by OD_600_ and centrifugation of a culture volume that would correspond to 1 mL of OD_600_ 1.0 culture. Metabolite extraction was performed based on the method of Overy et al. (2005). Cell pellets were re-suspended in 1 mL methanol:chloroform 1:1 (−20 °C) and vortexed to disrupt the pellet. Samples were then stored at −80 °C for 1 h before further disruption through vigorous shaking with cold chloroform:methanol (1:1) washed stainless steel ball bearings (2 mm diameter). Samples were again stored at −80 °C for 1 h before the addition of 400 μL ice-cold ultra high pure (UHP) water. Tubes were mixed by vortexing before centrifugation (15, 871×*g*, 1 min, 4 °C) whereafter the top aqueous phase (methanol plus water) containing mainly polar metabolites was decanted into a new cooled 1.5 mL plastic tube. Then 400 μL ice-cold UHP water was added to the chloroform (organic) phase containing mainly non-polar metabolites and centrifuged (15, 871×*g*, 1 min, 4 °C). The top aqueous phase was again decanted into the 1.5 mL plastic tube containing the previously harvested aqueous phase. Both aqueous and organic phases were then stored at −80 °C until analysis. A negative control of the above full harvesting and extraction procedure was carried but without the addition of bacteria. The metabolite profiles of these negative controls were outliers in the subsequent PCA analysis (data not shown).

### Electrospray ionisation time of flight mass spectrometry (ESI-TOF MS)

ESI-TOF MS was performed on a LCT spectrometer (Waters Ltd, Manchester, UK) based on the methods described in Davey et al. (2008) and Walker (2011). Data acquisition and processing was performed on MassLynx (version 4) to create centroid peak lists (*m/z* accurate to 4 decimal places vs. ion counts), which were then transferred to Microsoft Excel (Microsoft Corp, USA) as text files. The mass spectrometer was operated at a resolution of 4,000 (FWHM) at mass 200 *m/z* in positive and negative ion modes at a capillary voltage of 2,800 V (positive) and 2,500 V (negative), extraction cone at 3 V and sample cone at 20 V with a rangefinder lens voltage of 75 V chosen for detection of masses from 50 to 800 Da. Source temperature was 110 °C and desolvation temperature was 120 °C. Flow rates were 100 L h^−1^ for nebulisation and 400 L h^−1^ for desolvation. Spectra were collected in centroid mode at a rate of one spectrum s^−1^ (0.95 s scan time, 0.05 s interscan delay) with 180 summed over a 3-min period before being exported without background subtraction or smoothing.

Samples were either loaded using a syringe pump (Razel, Connecticut, USA) at a flow rate of 20 μL min^−1^ (organic phase) or loaded using an automated Waters 2695 Separations Module combining a HPLC pump and an autosampler (Waters, Hemel Hempstead, UK) (aqueous phase) with an inject volume of 100 μL at a flow rate of 50 μL min^−1^. A Lockspray™ interface was used to give an external standard and allow automated correction of mass measurements (5 ng μL^−1^ sulphadimethoxine giving a lockmass of 309.0653 or 311.0814 for negative and positive modes respectively). Samples were analysed in a randomised order to minimize effects of day-to-day machine variation.

### Data processing

For each sample run, the summation of 180 centroid mode spectra were exported from MassLynx data systems as text file peak lists (Accurate mass to 4 decimal places vs. ion count). These were imported into Microsoft Excel (Microsoft Corp, USA) and an in-house macro programme (created by Prof. Mike Burrell, Sheffield University, UK) used to compare the accurate masses of three technical replicate analyses of each sample. The accepted range to give the maximum number of peaks with minimum false positives was proposed by Overy et al. (2005) to be defined best by plotting the acceptable mass variance as a linear function of the *m/z* values. The same equations were used: positive mode, *y* < 0.00003*x* + 0.0033; for negative mode *y* < 0.00003*x* + 0.044 where *y* is the standard deviation of the three masses and *x* is the mean of the three masses. Those masses found to have a *y* value within acceptable limits had their mean accurate mass and mean response (as % total ion count in order to minimize sample–sample variation and normalize data sets) exported to a separate table. This methodology negated the need for a noise threshold and enabled true low intensity metabolite peaks to be kept. principal component analysis (PCA) was carried out using Pareto scaled 0.2 Da binned data sets in Simca-P v12.0 software (Umetrics, Sweden). Significance values of the ion counts between the samples were determined using two-tailed *t* tests.

### Putative metabolite identification

Preliminary identification of metabolites was performed through the comparison of monoisotopic masses likely to be present in extracts, including [M+H]^+^, [M−H]^−^ and [M+Na]^+^ against the list of metabolites in the biocyc database (http://biocyc.org/) and to a subset of metabolites within biocyc predicted to be present in *C. jejuni* NCTC 11168 (http://biocyc.org/CJEJ192222/class-tree?object=Compounds) based on the genome annotation, to an accuracy of 0.2 Da (Davey 2011).

## Results and discussion

### Direct injection ESI-TOF MS methodologies can distinguish *C. jejuni* metabolomic profiles

Methodology for both the quenching of metabolism and extraction of metabolites is still a controversial area and can require significant optimisation for individual organisms to yield reproducible data (Meyer et al. 2013). Many studies have looked at the effectiveness of quenching and extraction techniques through statistical methods (Bolten et al. 2007; Faijes et al. 2007). However, very few have been focussed on the ability to see biologically relevant metabolite changes within a non-targeted metabolomics analysis as a measure of quenching and extraction effectiveness. A variety of quenching techniques have been adopted for bacterial metabolomics, with the most popular methods being quenching in cold organic solvents, followed by centrifugation and extraction (Bolten et al. 2007) or rapid filtration based methods, although metabolite leakage problems are still an issue. Agar plate membrane filter culture techniques followed by rapid solvent quenching is one solution for these problems (Bennett et al. 2008).

In this study, a complex liquid medium (BHI-FCS) was used for bacterial cultivation to ensure as similar growth as possible of wild-type and mutant strains as well as maintaining activity of amino-acid catabolic routes. The use of a simple centrifugation step to collect the cells meant a methodology could be employed that resulted in no metabolite loss, through the freezing of whole cell pellets in liquid nitrogen (Bolten et al. 2007) before solvent extraction. Although even brief centrifugation may cause some metabolic perturbation, the effects should be minimised when directly comparing wild-type and mutant cell metabolomes, as both sample groups are treated identically. Similarly, washing steps were avoided to prevent metabolite loss but contamination of cell pellets with residual medium will be the same for each sample compared and medium-only controls were also run. Although the efficiency of extraction techniques is organism specific (Faijes et al. 2007; Meyer et al. 2013), comparative studies have shown cold methanol based methodologies to be among the most efficient for extracting a wide range of metabolites, in a broad spectrum of bacteria (Maharjan and Ferenci 2003; Park et al. 2012). For this reason a cold methanol:chloroform based extraction protocol was adopted here (adapted from Overy et al. (2005)).

**Fig. 1:**
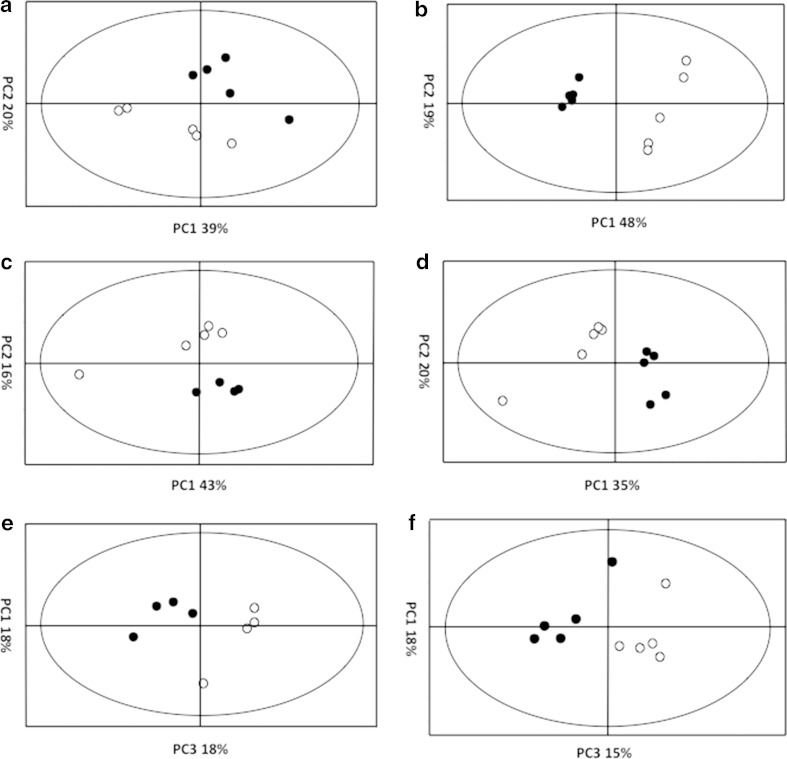
Principal component analysis (PCA) of *C. jejuni* NCTC 11168 and amino acid catabolism mutant strains. Metabolite levels in multiple replicates of *C. jejuni* NCTC 11168 and *aspB* strains were analysed by ESI-TOF MS in positive and negative ionisation mode and processed so that centroid *m/z* peaks were converted to text file peak lists and placed into 0.2 Da bins. Principal component analysis was performed on these bin lists. PCA plot **a** and **b** show analysis of positive and negative ionization datasets respectively for *C. jejuni* NCTC 11168 (*filled circles*) and *aspA* (*open circles*). PCA plot (**c**) and (**d**) show analysis of positive and negative ionization datasets respectively for *C. jejuni* NCTC 11168 (*filled circles*) and *aspB* (*open circles*). PCA plot (**e**) and (**f**) show analysis of positive and negative ionization datasets respectively for *C. jejuni* NCTC 11168 (*filled circles*) and *sdaA* (*open circles*). In all instances mutant strains were observed to group separately from their isogenic parent strain

Multiple replicates of *aspA, aspB* and *sdaA* mutant extracts were analysed by ESI-TOF MS alongside extracts from the *C. jejuni* NCTC 11168 isogenic parent strain in both negative ionisation mode (to focus on detecting carboxylic acids and alcohols) and positive ionization mode (to focus on detecting amines). Background noise was removed by selecting peaks whereby mass variance between three technical replicates fell within an accepted range and data was normalized to the total ion count before being separated into 0.2 Da mass unit bins (Online resources 1 and 2). PCA can clearly be seen to separate mutant profiles from their isogenic parent strain (Fig. 1). Positive ionization datasets, containing a larger number of ionization species, could be separated in a similar fashion to negative ionization datasets.

### Allocation of putative metabolite identities to the *C. jejuni* metabolome

Dunn et al. (2005) have previously shown DIMS methods to be capable of identifying metabolites of the same nominal but different monoisotopic mass. In our work, protocols were adapted to minimize problems of fragmentation and ion suppression as previously highlighted (Dunn et al. 2005) and putative metabolite identities were preferentially assigned to negative ionization data due to the existence of fewer likely ionic species [M−H]^−^. A full list of *m/*z bin allocations and the associated metabolite identities can be seen in Online resources 1–3.

It has been stated that bacterial metabolomes contain low numbers of metabolites, i.e. ~600 predicted for *E. coli* and *Saccharomyces cerevisiae* (Wang et al. 2006) and 467 in a recent metabolic model of *C. jejuni* metabolism (Metris et al. 2011). Clearly a reliance on genomic analysis to make predictions (Oliver et al. 1998; Metris et al. 2011) will underestimate metabolome complexity, with many enzymes likely to work on broader substrate ranges within the cell than generally accepted, and many genes with unknown metabolic roles. In our work, the large metabolite list from the biocyc database has been used to maximise allocated peak identities. However, this obviously may result in metabolites being allocated that do not lie within predicted metabolic routes of *C. jejuni.* We therefore also present metabolite tables in Online resources 1–3 based only on a comparison with predicted metabolites in *C. jejuni* NCTC 11168 as derived from the automated genome annotation. It is essential that putative metabolite allocations relating to changes of interest are thoroughly reviewed and should be confirmed in future work by other targeted assays.

### Putative metabolite identification in characterised metabolic mutants is consistent with known blocks in metabolism

**Fig. 2:**
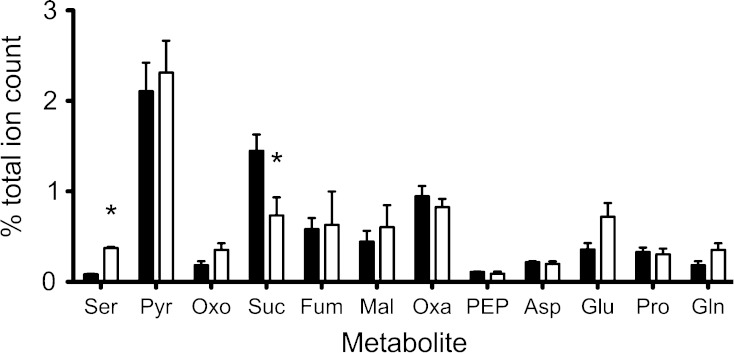
Key metabolite differences between *C. jejuni* NCTC 11168 and *sdaA* strains. Following ESI-TOF MS of five biological replicates in negative ionisation mode, *m/z* peaks were assigned putative metabolite identities to an accuracy of 0.2 Da for *C. jejuni* NCTC 11168 (*black bars*) and *sdaA* strains (*open bars*). Shown are those metabolites detected that are most closely related to amino-acid catabolism [*Ser* serine (104.00); *Pyr* pyruvate (87.00); *Oxo* 2-oxoglutarate (145.00); *Suc* Succinate (117.00); *Fum* Fumarate (115.00); *Mal* Malate (133.00); *Oxa* oxaloacetate (134.00); *PEP* phosphoenolpyruvate (167.00); *Asp* aspartate (132.00); *Glu* glutamate (146.00); *Pro* proline (115.00); *Gln* glutamine (145.00)] as a % of the total ion count. * *p* value <0.05 between *C. jejuni* NCTC 11168 and *sdaA* strain

Serine dehydratase catalyses the deamination of serine to form pyruvate and ammonium ions, in the first step of serine catabolism. In replicate extracts of the *sdaA* mutant compared to the wild-type, analyzed in negative ion ionisation mode, *m/z* 104.00, putatively corresponding to serine, was found to have undergone a 4.5 fold increase from 0.083 ± 0.016 to 0.37 ± 0.03 % of the total ion count with a *p* value of 8.2 × 10^−7^ (Fig. 2). Other key *m/z* values corresponding to metabolites of central metabolism within and connected with the citric-acid cycle were found to be highly similar with only *m/z* 117.00 (succinate) showing a statistically significant decrease in the mutant strain (Fig. 2). These results are consistent with the enzymatic block in the *sdaA* mutant resulting in a build-up of serine relative to wild-type. In total, however, we found only about 4.3 % of the total negative ion metabolite bins were significantly changed in the *sdaA* mutant (Table 1 and Online resource 1). The lack of other major central metabolite changes in this mutant is consistent with the only major effect on cell physiology being lack of growth on l-serine as C-source in minimal media (Velayudhan et al. 2004; Guccione et al. 2008). The fact that aspartate, proline and glutamate can still be utilised as carbon sources and that no growth defect is observed in complex media (Velayudhan et al. 2004) may explain why pyruvate levels are also maintained.

**Table 1:** Proportion of metabolite bins changed in each of the mutant strains analysed in this study, compared to the isogenic wild-type parent strain

Strain	Ionisation mode	Total metabolite count	Significantly changed	% Metabolites changed
*sdaA*	−	2,167	94	4.34
*aspB*	−	2,171	135	6.22
*aspA*	−	1,568	278	17.73
*cj0150c*	−	2,060	267	12.96
*sdaA*	+	1,474	72	4.88
*aspB*	+	1,532	135	8.81
*aspA*	+	1,568	223	14.22

The table shows how many bins have a value in at least one replicate and how many of those are significant at *p* < 0.05 when comparing each mutant with the wild-type strain

**Fig. 3:**
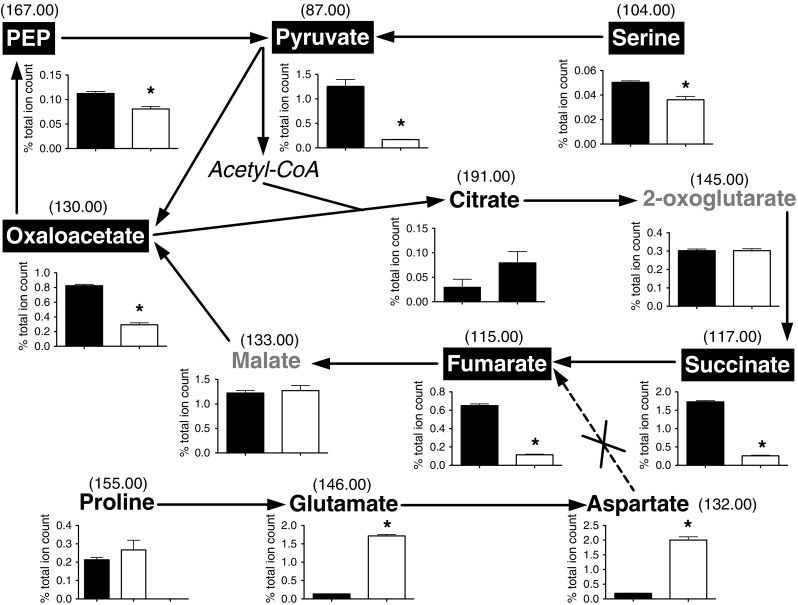
The effect of an *aspA* null mutation on central carbon metabolism. Metabolites in black typeface show an increase in relative abundance in the *aspA* mutant; white typeface a decrease in relative abundance; *grey* typeface no change and *italicized* typeface, not detected in either strain. *p value <0.05 between the *C. jejuni* NCTC 11168 (*black bars*) and *aspA* strain metabolite levels (*open bars*), displayed as a % of the total ion count with bin values depicted above. Aspartate and glutamate are 10.5 and 12.6 fold increased respectively in the *aspA* strain compared to the wild-type. Most central metabolites are lower in relative abundance in the *aspA* strain, exceptions being 2-oxoglutarate, a glutamate precursor, malate and citrate

The metabolite profile of the *aspA* mutant was found to be distinctly different, with significant changes in the relative abundance of a much larger number of metabolites (Table 1 and Online resource 2), indicating a large-scale disruption to central as well as more peripheral metabolism. Aspartase catalyses the deamination of aspartate to fumarate plus ammonium ions and analysis of the metabolite levels show a relative accumulation of *m/z* 146.00 (glutamate) and *m/z* 132.00 (aspartate), and significantly reduced levels of *m/z* 115.00 (fumarate) and several putative TCA cycle metabolites, consistent with the metabolic lesion in this mutant (Fig. 3). An interesting exception is *m/z* 145.00 (2-oxoglutarate) (0.30 ± 0.02 and 0.30 ± 0.02 % total ion count in NCTC 11168 and *aspA* mutant respectively), but this cannot be distinguished from glutamine from our analysis. The many metabolite changes are consistent with the known phenotype of the *aspA* mutant, which cannot grow on aspartate, glutamate or proline and has a reduced growth rate compared to the wild-type, even in complex media (Guccione et al. 2008).

**Fig. 4:**
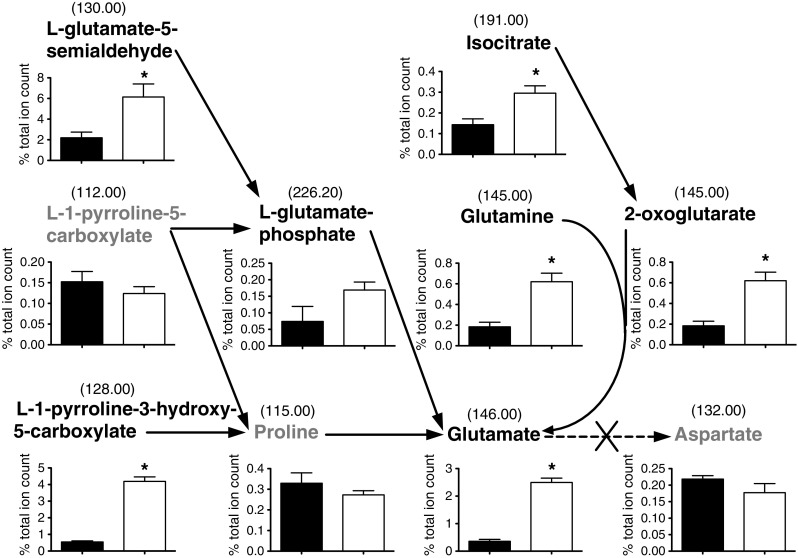
The effect of an *aspB* null mutation on glutamate metabolism. Having putatively identified metabolites from ESI-TOF MS negative ionisation mode the relative metabolite levels were analysed related to the lesion in metabolism caused by *aspB* mutation (*black cross*). Metabolites in black typeface show an increase in relative abundance in the *aspB* strain and in *grey* typeface a decrease. *p value < 0.05 between the *C. jejuni* NCTC 11168 (*black bars*) and *aspA* strain (*white bars*) metabolite levels, displayed as a % of the total ion count. Glutamate can be seen to have undergone a significant sevenfold increase and aspartate a slight decrease in the *aspB* mutant. Many metabolites linked to glutamate can be seen to have undergone an increase in the *aspB* strain

AspB in *C. jejuni* is encoded by *cj0762c* and is essential for growth on glutamate, as it converts glutamate to aspartate using oxaloacetate as the amino-acceptor. In the *aspB* mutant a significant accumulation of *m/z* 146.00 (glutamate) is observed preceding the metabolic lesion as well as a significant increase in *m/z* 145.00 (2-oxoglutarate). Compared to the *aspA* mutant, fewer metabolites overall were significantly changed (Table 1 and Online resources 1 and 2). However, several glutamate precursors in various metabolic pathways have undergone significant changes (Fig. 4), which are not readily interpretable but might, for example, result from changes in enzyme allosteric control.

Analyses of these previously characterised metabolic mutants clearly shows that a simple ESI-TOF direct injection method is capable of detecting the corresponding metabolic lesion and provides unique information about how the mutation is affecting the wider metabolome (Online resources 1 and 2). Removal of AspA (by mutation) from the highly interconnected cellular enzyme network clearly has a “ripple effect” that spreads far beyond the mutant metabolic lesion. This has been observed in some other bacterial metabolic mutant studies [for a recent example see Lee et al. (2014)]. The observed metabolite changes will be the combined result of many processes e.g. altered allosteric control of enzyme activities by key metabolites and changed expression of enzyme encoding genes as a result of altered activity of regulatory proteins. It would therefore be highly informative to correlate mutant metabolomic data with transcriptomic and proteomic data to provide an integrated view that might give insights into the types of mechanisms involved.

### Analysis of a *cj0150c* mutant reveals a possible role for Cj0150 as a cystathionine β lyase

**Fig. 5:**
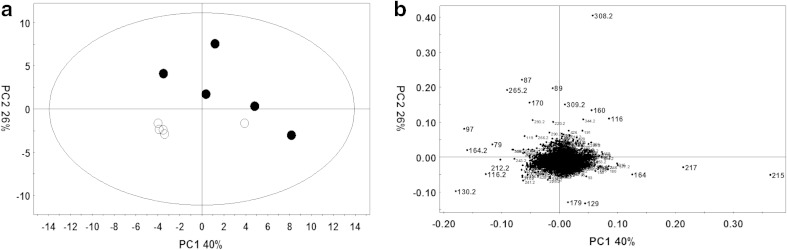
Principal component analysis of *C. jejuni* NCTC 11168 and *cj0150c* strains from negative ionisation mode metabolite profiles. Metabolite levels in multiple replicates of *C. jejuni* NCTC 11168 and *cj0150c* strains were analysed by ESI-TOF MS in negative ionisation mode and processed so that centroid *m/z* peaks were converted to text file peak lists and placed into 0.2 Da bins. Principal component analysis was performed on these bin lists. PCA plot (**a**) shows analysis of negative ionisation data with principal component 1 (PC1) against principal component 2 (PC2). *C. jejuni* NCTC 11168 and *cj0150c* are represented as *closed circles* and *open circles* respectively with the corresponding score loading plot shown in (**b**)

**Fig. 6:**
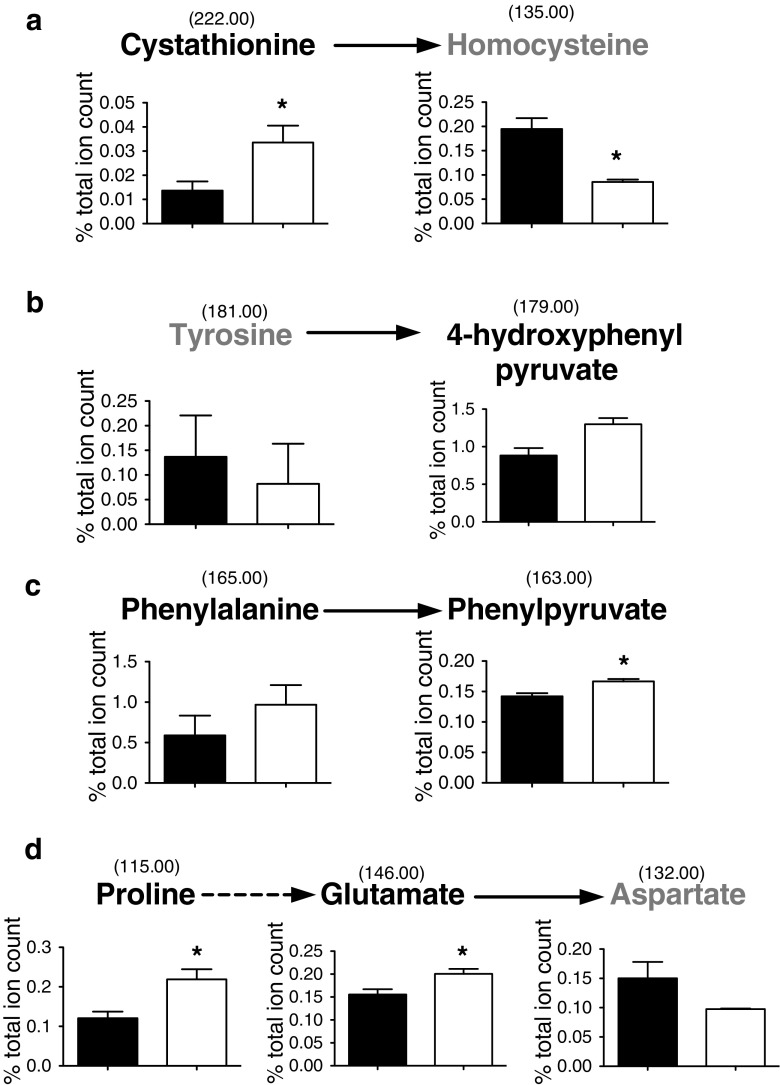
Significant metabolite changes for potential aminotransferase activities in the *cj0150c* strain. Significant changes were observed in metabolites related to: **a** cystathionine β lyase activity and metabolites related to general type I aminotransferase activity such as **b** tyrosine aminotransferase **c** phenylalanine aminotransferase and **d** aspartase activity. Wild type and *cj0150c* are shown as *black* and *white* bars respectively. *p value <0.05, an *arrow* represents an aminotransferase reaction and bin values for each metabolite seen above. Metabolites in *black* typeface show an increase in relative abundance in the *cj0150* strain and in *grey* typeface, a decrease

In contrast to SdaA, AspA and AspB, Cj0150 is an enzyme of unknown function. Sequence analysis shows that it is a member of the amino-acid aminotransferase type I family (Guccione et al. 2008). These enzymes are known to be active with a wide range of substrates but it is not possible from sequence similarities alone to ascribe a physiological function to them. Purified recombinant Cj0150 has previously been shown to have aspartate:glutamate aminotransferase activity (Guccione et al. 2008), but this was not thought to be physiologically significant as a *cj0150c* mutant strain shows no growth defect on glutamate, whereas a *cj0762c* (*aspB*) mutant is completely unable to grow on or utilise glutamate or proline as a carbon source (Guccione et al. 2008). Here, we compared the metabolic fingerprints of the isogenic wild-type and *cj0150c* mutant by DIMS. PCA analysis of the data show ESI-TOF MS based metabolomics is capable of distinguishing the wild-type and mutant strain, even though the latter has no obvious growth or other known phenotype (Fig. 5). Analysis of the changes in the relative abundance of the putatively identified metabolites, suggests that although the *cj0150c* mutant is capable of growth on glutamate or proline, it is in fact providing some level of aspartate:glutamate aminotransferase activity in vivo as evidenced by a small but significant change in the ratio of glutamate and aspartate in the wild-type and mutant strains (Fig. 6). Most interestingly, we noted larger significant changes in two metabolites that we putatively identified as cystathionine and homocysteine. Changes in cystathionine and homocysteine levels would be consistent with Cj0150 being a cystathionine β lyase. Cystathionine β lyases are PLP dependent enzymes that function in the transulphuration pathway of methionine biosynthesis, converting cystathionine to homocysteine via an α/β elimination reaction (Auger et al. 2005). Significantly, Guccione et al. (2008) carried out a phylogenetic analysis of type I aminotransferases and found that Cj0150 clusters within the IΩ subgroup, along with enzymes experimentally found to possess cystathionine β lyase activity (Auger et al. 2005; Chu et al. 1995). In addition, the gene immediately downstream of *cj0150c* in strain NCTC 11168 encodes homoserine dehydrogenase; an enzyme that functions to produce homoserine from aspartate semialdehyde, an intermediate in the methionine biosynthetic pathway. It is important to note that *C. jejuni* does contain an annotated *metC* gene (*cj1393*), the major cystathionine β lyase known to perform this function (Lodha et al. 2010), and that methionine levels were maintained in the *cj0150c* strain. For this reason it is difficult to confidently predict whether the cystathionine β lyase activity of Cj0150 would be physiologically relevant for methionine biosynthesis, but determination of the kinetics of this activity for purified Cj0150 is clearly warranted. Although some changes in other putative metabolites related to aminotransferase activity were seen (tyrosine aminotransferase, phenylalanine aminotransferase; Fig. 6 and Online resource 3) the extent of the changes were less convincing, compared to those discussed above.

## Concluding remarks

We have shown that DIMS fingerprints can separate metabolomic profiles of metabolic mutants of *C. jejuni* and give insights into the degree to which the metabolome is changed by specific enzymatic blocks. Although the ESI-TOF MS based metabolomics method used here suffers from a lack of mass accuracy its high sensitivity and high throughput make it an ideal strategy for the initial analysis of differences in a large number of cellular metabolites in mutant screens. Information from the DIMS fingerprint of a mutant with no obvious phenotype has also been used here to suggest an in vivo activity for the Cj0150 enzyme.

## Electronic supplementary material

Below is the link to the electronic supplementary material.

Online resource 1. Excel spread sheet showing 0.2 Da mass unit bin centres of peaks normalized to total ion count for *C. jejuni* NCTC 11168, *sdaA* and *aspB* strains run in positive and negative ionisation modes. Putative metabolite allocations based on the full and *Campylobacter jejuni jejuni* NCTC 11168 specific biocyc (www.biocyc.com) compounds lists and preliminary data analysis are also shown (XLS 8510 kb)

Online resource 2. Excel spread sheet showing 0.2 Da mass unit bin centres of peaks normalized to total ion count for *C. jejuni* NCTC 11168 and *aspA* strains run in positive and negative ionisation modes. Putative metabolite allocations based on the full and *Campylobacter jejuni jejuni* NCTC 11168 specific biocyc (www.biocyc.com) compounds lists and preliminary data analysis are also shown (XLS 6040 kb)

Online resource 3. Excel spread sheet showing 0.2 Da mass unit bin centres of peaks normalized to total ion count for *C. jejuni* NCTC 11168 and *cj0150c* strains run in negative ionisation mode. Putative metabolite allocations based on the full and *Campylobacter jejuni jejuni* NCTC 11168 specific biocyc (www.biocyc.com) compounds lists and preliminary data analysis are also shown (XLS 2486 kb)
